# Active Moss Biomonitoring of Mercury in the Mine-Polluted Area of Abbadia San Salvatore (Mt. Amiata, Central Italy)

**DOI:** 10.3390/toxics13010002

**Published:** 2024-12-24

**Authors:** Federica Meloni, Sergio Calabrese, Orlando Vaselli, Francesco Capecchiacci, Francesco Ciani, Lorenzo Brusca, Sergio Bellomo, Walter D’Alessandro, Kyriaki Daskalopoulou, Stefania Venturi, Barbara Nisi, Daniele Rappuoli, Franco Tassi, Jacopo Cabassi

**Affiliations:** 1 Department of Earth Sciences, University of Florence, Via La Pira 4, 50121 Florence, Italy; federica.meloni@unifi.it (F.M.);; 2 CNR-IGG Institute of Geosciences and Earth Resources, Via La Pira 4, 50121 Florence, Italy; 3 Department of Earth and Marine Sciences (DiSTeM), University of Palermo, Via Archirafi 36, 90123 Palermo, Italy; 4 Istituto Nazionale di Geofisica e Vulcanologia, Palermo Unit, Via Ugo La Malfa 153, 90146 Palermo, Italy; 5 Istituto Nazionale di Geofisica e Vulcanologia, Bologna Unit, Viale Carlo Berti Pichat 6/2, 40127 Bologna, Italy; 6 Istituto Nazionale di Geofisica e Vulcanologia, Naples Unit, Osservatorio Vesuviano, Via Diocleziano 328, 80125 Naples, Italy; 7 Institute of Geosciences, University of Potsdam, Karl-Liebknecht-Str. 24-25, 14476 Potsdam Golm, Germany; 8 GeoForschungs Zentrum, Physics of Earthquakes and Volcanoes, Helmholtzstraße 6/7, 14467 Potsdam, Germany; 9 Unione dei Comuni Amiata Val d’Orcia, Unità di Bonifica, Via Grossetana 209, 53025 Piancastagnaio, Italy; 10 Parco Museo Minerario di Abbadia San Salvatore, Via Suor Gemma 1, 53021 Abbadia San Salvatore, Italy; 11 Istituto Nazionale di Geofisica e Vulcanologia, Roma1 Unit, Via di Vigna Murata 605, 00143 Rome, Italy

**Keywords:** moss bags, biomonitoring, GEM, mercury, pollution, mining

## Abstract

Active biomonitoring of mercury (Hg) using non-indigenous moss bags was performed for the first time within and around the former Hg mining area of Abbadia San Salvatore (Mt. Amiata, central Italy). The purpose was to discern the Hg spatial distribution, identify the most polluted areas, and evaluate the impacts of dry and wet deposition on mosses. The exposed moss bags consisted of a mixture of *Sphagnum fuscum* and *Sphagnum tenellum* from an external uncontaminated area. In each site, two different types of moss bags, one uncovered (to account for the wet + dry deposition) and one covered (to evaluate the dry deposition), were exposed. The behavior of arsenic (As) and antimony (Sb) in the mosses was investigated to assess the potential relationship with Hg. GEM (Gaseous Elemental Mercury) concentrations were also measured at the same sites where the mosses were exposed, although only as a reference in the initial stages of biomonitoring. The results revealed that the main Hg emissions sources were associated with the former mining area of Abbadia San Salvatore, in agreement with the measured GEM concentrations, while arsenic and antimony were related to soil enriched in As-Sb waste material. The three elements registered higher concentrations in uncovered mosses with respect to the covered ones, i.e., wet deposition was the key factor for their accumulation on the uncovered mosses, while dry deposition was especially important for the covered samples in the mining area. Hg was accumulated in the mosses via GEM adsorption, uptake of particulate Hg, and precipitation via raindrops/snowfall, with almost no loss and without post-deposition volatilization. The results testified that the chosen biomonitoring technique was an extremely useful tool for understanding Hg transport and fate in a contaminated area.

## 1. Introduction

Mercury dispersion from anthropized, e.g., [1,2,3] and natural areas, e.g., [4,5,6], has an immediate and pervasive impact on the surrounding environment due to the high toxicity of this element to both ecosystems and humans [7,8,9]. In particular, areas of active or abandoned Hg mining sites notably contribute to the dispersion of Hg into the environment from either metallurgical processes, mining structures and complexes, or contaminated soils and industrial wastes, e.g., [10,11]. It is therefore becoming increasingly urgent to inventory, control, and monitor mercury emissions using specific techniques, as strongly recommended by the United Nations Minamata Convention on Mercury and the Global Mercury Partnership [12,13,14] and the European Air Quality Directives [15].

One of the most sensitive, reliable, cost-effective, and easy-to-use monitoring methods for all the metals that can be mobilized by a pollutant source is biomonitoring, e.g., [16], i.e., the use of organisms and biomaterials (bioindicators) to obtain information and track changes on the characteristics and conditions of the environment [17]. In particular, the moss bag technique, based on the exposition to air of moss samples in mesh bags, is the most common biomonitoring method for pollutants in the atmosphere [18]. The principle of this technique is based on the passive accumulation of atmospheric elements by mosses through both dry and wet deposition [19]. Bryophytes, like mosses, lack a well-defined root system, thereby absorbing both nutrients and other compounds almost exclusively from the atmosphere. Moreover, thanks to their high specific surface, they are able to uptake gases and atmospheric particles from the air [20]. Mosses are thus widely used to monitor mercury (Hg) and/or other trace elements in the atmosphere, in urban and industrial environments [21,22], in volcanic or geothermal areas [17,23], in indoor environments [24], and close to mining plants [25].

In this paper, we present the first attempt at Hg active biomonitoring using non-indigenous moss bags within and around the former Hg mining area of Abbadia San Salvatore (ASS) (Mt. Amiata, central Italy), i.e., one of the world’s leading areas for cinnabar exploitation and liquid mercury production for about a century between ‘800 and ‘900, e.g., [26,27]. Concentrations of GEM (Gaseous Elemental Mercury), which is the most abundant gaseous mercury species in the atmosphere, e.g., [28], were also measured using a highly sensitive instrument (Lumex RA-915M, Lumex Instruments, British Columbia, Canada) in the same places where mosses were exposed, although as a reference measurement of air contamination in the initial phase of biomonitoring. The main aims of this study are to (i) describe the Hg spatial distribution in the air based on the moss bag technique, (ii) identify the most polluted areas, and (iii) evaluate the different impacts of dry and wet deposition on mosses. As arsenic (As) and antimony (Sb) sulfides, i.e., mainly realgar and stibnite [29,30], were also found in the Hg-mineralized area of ASS in association with the cinnabar mineralization, we additionally investigate the behavior of As and Sb in the mosses and their potential relationship with Hg.

## 2. Study Area

**Figure 1:**
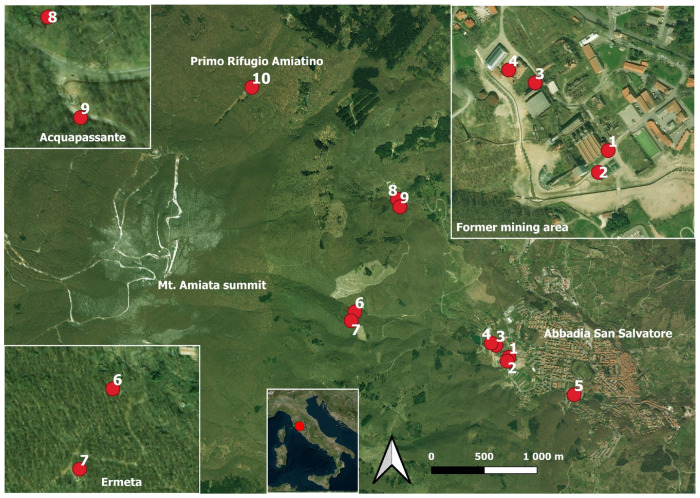
Study area and sampling sites location.

The study area is located inside and nearby ASS (Figure 1), i.e., the principal site of mining and liquid Hg production of the Mt. Amiata mercury district [26,27]. The numerous buildings, structures, and open spaces of the former mining area are affected by Hg contamination and dispersion. This has resulted in high GEM contents (up to >50,000 ng/m^3^; [26]), high values of total and leachable Hg in the synthetic materials [27] and soils [30,31], and remarkable concentrations of dissolved Hg in water samples [32].

Consequently, the mining complex was divided by the Municipality of ASS into seven different units, with the most contaminated constructions pertaining to Unit 6 (please refer to [26,27,31]). The entire site is currently under remediation in order to be recovered as a historic museum and mining park and to be preserved as an archaeometallurgical treasure. The environmental impact of such a mercury mining activity on both geosphere and biosphere has been studied in detail by many authors, e.g., [33,34], being also ascribable to mining wells drilled above the mining area of ASS (Ermeta and Acquapassante: 1079 and 1048 m a.s.l.; [35]), where the periodic monitoring of H_2_S, CO_2_, and GEM concentrations has been carried out. In this context, studies with different biomonitoring tools (i.e., tree barks, rings, leaves and cores, lichens, native mosses, and plants) were also performed over the years, providing considerable insights into the distribution and environmental impact of mercury, e.g., [36,37,38,39,40,41].

## 3. Materials and Methods

Mercury, As, and Sb accumulation through moss bags was performed in an approximately two-month measurement campaign, at Unit 6 of the former ASS mine (~870 ÷ 890 m a.s.l.) and the surrounding areas of the mining chimneys of Ermeta and Acquapassante [35], for a total of 10 fixed measuring points (Figure 1; Table 1). Measurement sites positioned in distant areas (Primo Rifugio Amiatino: ~1280 m a.s.l.; tennis club at the ASS urban center: ~840 m a.s.l.) were also included to evaluate the extent of the pollutants’ dispersion. The mosses exposure period lasted from October to December 2013, i.e., when the operations in the former mining area mainly consisted of moving soil and waste materials and before the start of the remediation activities on the main buildings and edifices.

The exposed moss bags consisted of a mixture of *Sphagnum fuscum* (Schimp.) H.Klinggr and *Sphagnum tenellum* Sw. ex Willd, from an uncontaminated area in Sweden, which is characterized by excellent water retention, surface wettability, and cation exchange capacity [17,18]. The mosses were repeatedly washed with MilliQ water (approximately 18.3 MΩ/cm) to ensure low initial levels of trace elements and then dried at room temperature under a laminar hood. About 2 g of moss were then packaged in nylon bags (previously washed in a slightly acidic solution) with a 2 mm mesh to form a sphere of about 5 cm in diameter. Aliquots of moss were separated before and after cleaning procedures to evaluate their effect and to obtain background concentrations before field exposure (blank moss). Once in the field, at each measurement point, a wooden pole, about 1.5 m high, was driven into the ground, and moss bags were attached by means of nylon ropes affixed to a wooden rod mounted on the top of the pole [17]. In each site, one moss bag was exposed directly to the air (*uncovered-U*) to account for the bulk deposition (wet + dry), while a second bag was covered with plastic cup protection (*covered-C*) to exclusively evaluate the impact of dry deposition.

Simultaneously with the installation of the moss bags and only for a very limited time corresponding to the early stages of biomonitoring, the GEM concentrations at each site were also measured using an instrument (Lumex RA-915M Mercury Analyzer, Lumex Instruments, BC, Canada) set at continuous acquisition in datalogger mode. The instrument is based on differential atomic absorption spectrometry using high-frequency modulation of light polarization (ZAAS-HFM) [42], thus being able to continuously measure GEM concentrations (range: 2 ÷ 50,000 ng/m^3^) at high frequency (about 1 Hz) in real-time. The accuracy of the method is 20% [43], while a zero-correction system continuously checks the baseline during sampling by using an internal calibration cell.

After about a two-month exposure period, the moss bags were packed in polypropylene containers and were transported to the laboratory of the DiSTeM—University of Palermo. There, the mosses were removed from the bags, dried in an oven at 40 °C, weighted, and powdered by a planetary ball mill system equipped with agate jars to avoid contamination. The powder was split into two homogeneous sub-samples for (a) mercury (Hg_tot_) determination via Cold Vapor Atomic Absorption Spectrometry (CV-AAS) and (b) microwave digestion and Inductively Coupled Plasma Mass Spectrometry (ICP-MS) analysis, respectively. The two sub-samples were once again dried at 40 °C for 24 h and weighed on an analytical balance before chemical analyses. All the analyses were performed at INGV-Palermo laboratories.

(a) Hg_tot_ was analyzed with a Hydra-C Mercury Analyzer instrument (Teledyne Instruments Leeman Labs Inc., Hudson, New Hampshire, USA) based on the 7473 U.S. EPA method [44]. This method allows the determination of the Hg content by operating directly on the solid sample, avoiding losses or contamination. Mercury is released from the matrix by thermal decomposition, oxidized and separated from the other components, and subsequently trapped in a gold amalgam from which it is determined. Each sample was analyzed 5 times to verify the analytical reproducibility (SD ranging from 5 to 10%). Median blank values were subtracted from the median values of the samples;
(b) Arsenic and Sb were analyzed by ICP-MS (Agilent 7500-ce, Agilent, Santa Clara, CA, USA) after Closed-Vessel Acid Digestion of c.a. 0.25 g of dry powder in a microwave oven (CEM MARS Xpress, CEM Corporation, Matthews, NC, United States) using Teflon vessels with 3 mL concentrated HNO_3_ (ultrapure grade 65%), 2 mL H_2_O_2_ (ultrapure 39%) and 5 mL of MilliQ water with HNO_3_:H_2_O_2_ (2:1 ratio) [17]. The reliability and accuracy of As and Sb results were checked by analyzing four certified reference materials (certified leaves: NIST 1515, NCS DC 73,349, NCS DC 73,350, and NCS DC 73,351), and quality control was assessed by comparing obtained results with the certified values. The recovery values, i.e., the difference in percentage between measured and certified values for each element, were around 100 ± 10%. The same technique was also applied to determine the vanadium content that was then used as a reference metal in the calculation of the Hg, As, and Sb Enrichment Factor (EF, i.e., a parameter to evaluate the origin of the elements trapped by the mosses; cf. Section 4).

## 4. Results

Table 1 shows the geographical position and altitude of the fixed points where the moss bags were installed and their exposure times. The main data (in ng/g) related to the Hg, As, and Sb contents are listed in Table 2, subdividing between covered and uncovered (C and U, respectively) mosses and using a non-sampled (blank) moss as a reference. The 3U moss has been lost during sampling operations. For Hg, five replicates of each sample were performed as required by the used method; hence, summary statistical parameters (mean, median, standard deviation, maximum, and minimum value) are reported in Table 2.

**Figure 2:**
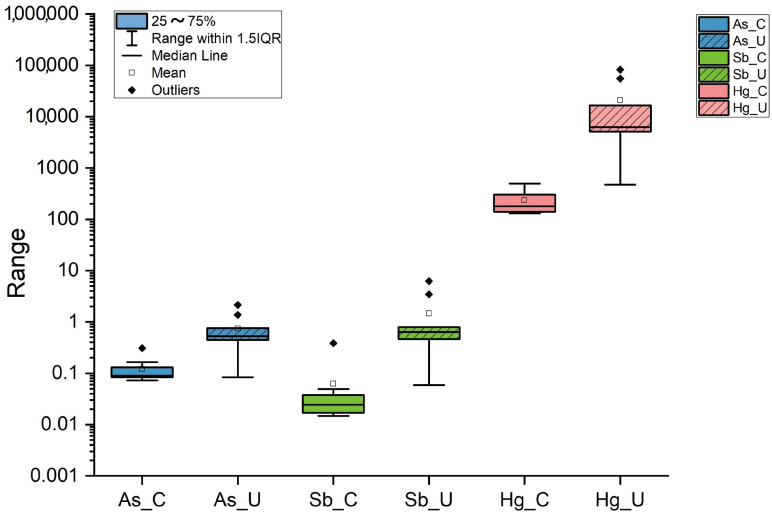
Boxplot of As, Sb, and Hg (in ng/g) in covered (C) and uncovered (U) mosses.

The Hg concentrations of the mosses directly exposed to the atmospheric agents (uncovered) were higher than those measured in the covered ones. The minimum, median, mean, maximum and standard deviation Hg values for covered mosses varied from 101 to 323 ng/g, from 130 to 497 ng/g, from 122 to 476 ng/g, from 136 to 653 ng/g and from 8.8 to 154 ng/g, respectively, while for uncovered mosses they ranged from 209 to 54,092 ng/g, from 473 to 82,317 ng/g, from 535 to 75,893 ng/g, from 923 to 133,842 ng/g and from 280 to 37,674 ng/g, respectively (Table 2). The highest values of As and Sb in covered mosses were 307 (1C moss) and 384 ng/g (4C moss), respectively, while the lowest ones were 72 (2C moss) and 15 ng/g (6C moss), respectively. On the other hand, the highest contents of As and Sb in uncovered mosses were 2147 and 6243 ng/g (1U moss), respectively, while the lowest ones (84 and 59 ng/g, respectively) were related to 5U moss (Table 2). Like Hg, As and Sb registered higher concentrations in uncovered mosses with respect to the covered ones (Figure 2), with the only exception being documented at site 5 for As.

Table 3 displays the GEM concentration measurements (5698 in total) performed at each point using the Lumex in datalogger mode (one-second acquisition time), providing insights, albeit partial, into Hg air contamination in the preliminary stages of biomonitoring. The GEM minimum, median, mean, maximum, and standard deviation concentrations were from 10 to 709 ng/m^3^, from 16 to 4785 ng/m^3^, from 17 to 5325 ng/m^3^, from 31 to 19,927 ng/m^3^, and from 2.6 to 3589 ng/m^3^, respectively.

**Figure 3:**
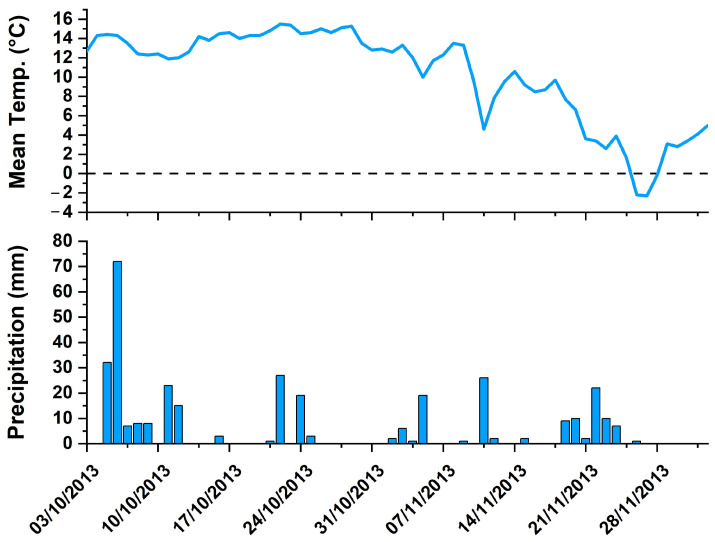
The average temperature (°C) and precipitation (mm) trend from the ASS weather station TOS07000001 (Tuscany Regional Hydrological Service, www.sir.toscana.it) during the study period. The dashed line indicates the temperature of 0 °C.

During the study period, the maximum, minimum, and mean temperature values were from 20.6 to −0.1 °C, from 13.3 to −4.2 °C, and from 15.5 to −2.3 °C, respectively. The precipitation varied from 0 to 72 mm, with a mean value of 5.5 mm. The full data are reported in Table S1, while in Figure 3, the average temperature trend (in °C) and the bar diagram representing the precipitation (in mm) are shown. All meteorological data were taken from the ASS weather station TOS07000001 (coordinates: UTM E 717954, UTM N 4752539; altitude: 855 m; Tuscany Regional Hydrological Service, www.sir.toscana.it (accessed on 14 October 2024)), close to the fixed points of moss bags exposure.

As reported by Calabrese et al. [17] and Kosior et al. [45], the accumulation value of the elements within mosses can be calculated by using the relative accumulation factor (RAF) formula and the median value of each moss and the blank (for Hg), or the value of each moss and the blank (for As and Sb):

RAF_x_ = [(C_x_)_exposed moss_ − (C_x_)_blank moss_]/(C_x_)_blank moss_ (1)

where RAF_X_ is the relative accumulation factor for each element X, and C_x_ is the concentration of each element in exposed and not exposed (blank) mosses. The RAF values for Hg, along with those of As and Sb for covered and uncovered mosses, are reported in Table 4. Accordingly, uncovered mosses inside the former mining area showed the highest RAF values (up to 648, 35, and 479 for Hg, As, and Sb, respectively), while the lowest RAF values corresponded to the covered mosses outside the former mining area for Hg and Sb, i.e., 8C moss (0.5 RAF) and 6C moss (0.1 RAF), respectively, and inside the former mining area (2C moss, 0.2 RAF) for As.

According to many authors, e.g., [46,47], the Enrichment Factor (EF) may be calculated through Equation (2) to evaluate whether the chalcophile elements trapped by the mosses are of anthropogenic or natural inputs:

EF = (C/R)_mosses_/(C/R)_soil_ (2)

where C is the concentration of relative metals in mosses and soil, respectively, while R is the concentration of a reference metal in mosses and soil, respectively. In this case, V was selected as a reference metal as it is considered a relatively immobile element in soil, e.g., [48].

The element concentration in soils is rather variable from region to region, being affected by many factors such as bedrock, climate, topography, physicochemical conditions, microbial activity, and vegetation, e.g., [49]. For this reason, this study considered the local geochemical baseline values of Hg, As, and V for topsoil reported by Meloni et al. [30], i.e., 21.2, 85.1, and 104.2 mg/kg, respectively. Concerning antimony, the soil concentrations were found to be below the Italian limit (Legislative Decree 152/06) for public green areas [30]. Therefore, the Italian limit of 10 mg/kg was chosen as the reference value for Sb.

On this basis, the calculated EF values can be divided into four classes according to the different enrichment levels in heavy metals [47,50,51]: (i) EF ≤ 2, no significant enrichment of the element; (ii) 2 ≤ EF ≤ 6, slight enrichment; (iii) 6 ≤ EF ≤ 10, moderate enrichment; and (iv) EF ≥ 10, strong enrichment.

The EF values of Hg, As, and Sb in the mosses are reported in Table 5. Similarly to RAF, the highest EF values are generally related to the uncovered mosses, especially for the moss bags collected inside the former ASS mining area (sites from 1 to 4; Figure 1; Table 1), with the only exception of site 8 for Sb. The covered and uncovered mosses have EF values ≤ 2 for As and Sb, with the only exception of 1U moss for both As (4.03) and Sb (11.7) and 2U moss for Sb (5.80). Hg presents high EF values in uncovered mosses (up to 103,504 for 1U moss), while only one covered moss (i.e., 1C) has EF > 2 (2.23), the others being ≤ 2. 1U and 2U are both located in the southernmost sector of the ASS mine.

## 5. Discussion

**Figure 4:**
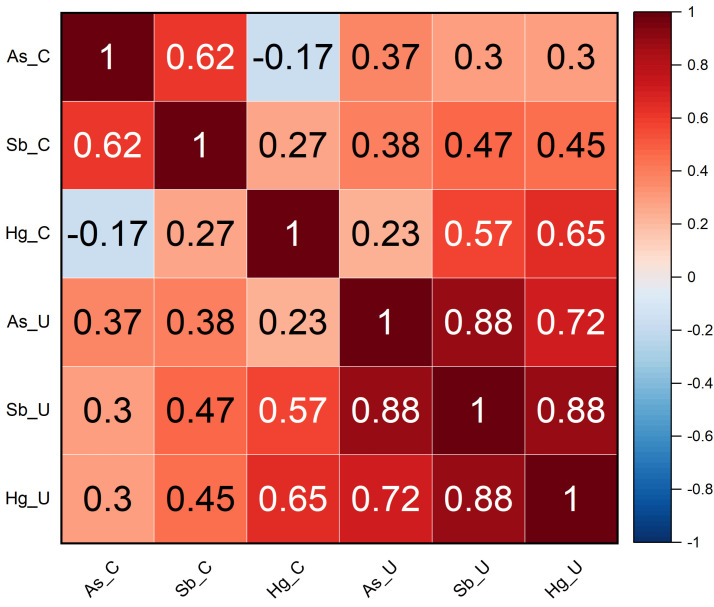
Correlation matrix of As, Sb, and Hg in covered (C) and uncovered (U) mosses. The number inside each cell represents Spearman’s correlation coefficient. See the text for further details.

In the great majority of cases, Hg, As, and Sb contamination is affecting the uncovered mosses. In particular, the sites located within the former and highly contaminated ASS mine, i.e., 1 and 2 (near the building that houses the old furnaces; Figure 1) and 4 (in front of the old mechanical workshop building), reached the maximum RAF values for covered (2C for Hg, 1C for As, and 4C for Sb) and uncovered (4U for Hg, 1U for As and Sb) samples (Table 4), and the highest EF values (1C and 1U for Hg and As, 4C and 1U for Sb, Table 5), thus proving that mosses accurately reflect the spatial distribution of the contamination, especially for Hg.

The potential relationship among the concentrations of the three elements (As, Sb, and Hg) trapped in, respectively, covered and uncovered mosses was tested using a correlation matrix (Figure 4). This was created using Spearman’s correlation coefficient (*p*-value < 0.05), which is the most robust against outliers [52]. In covered mosses, a good correlation between As and Sb (0.62) seems to point out the same origin for these two elements, while they have a poor correlation with Hg (−0.17 and 0.27, respectively), testifying that a different contamination source governs its accumulation on mosses. One possible explanation pertains to the sites where they were positioned, which were characterized by a different soil cover. It is indeed to be pointed out that As-Sb-rich post-roasting waste material from other Mt. Amiata Hg mines was stored in the southern part of Sector 6 [31,32]. The uncovered mosses show a relatively good correlation (0.88) between As and Sb and a significant positive correlation of the two elements with Hg (0.72 and 0.88, respectively), suggesting a strong influence of wet deposition on the accumulation of the three elements on mosses. This is also reflected in the observed poor correlation between As_C and As_U and between Sb_C and Sb_U, respectively. On the contrary, the good correlation between Hg in the covered and uncovered mosses underlines a unique source of Hg in the environment concerning the former mining activities.

**Figure 5:**
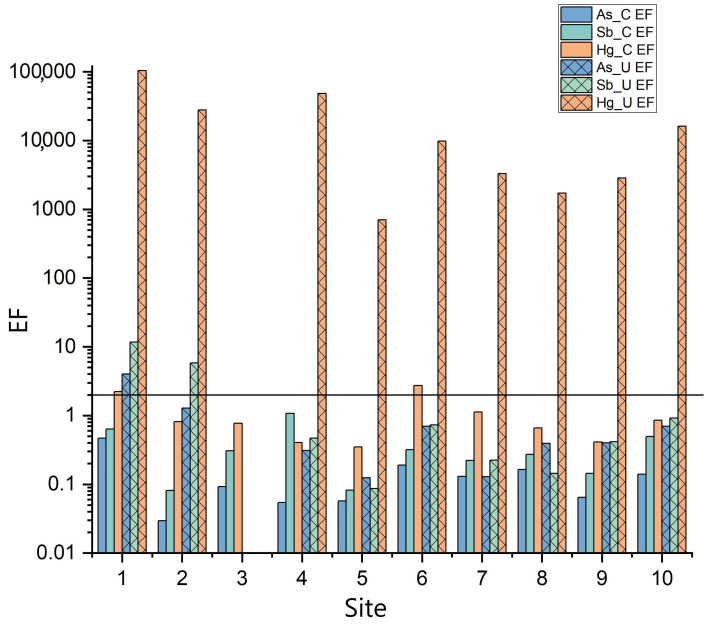
Bar diagram of the Enrichment Factor (EF) values of As, Sb, and Hg in covered (C) and uncovered (U) mosses divided according to the sampling sites. The black line represents EF = 2, i.e., the value above which enrichment in heavy metals occurs.

The EF values (Table 5 and Figure 5) show that for As and Sb, the covered and uncovered mosses do not present any enrichment (EF ≤ 2). According to Zarazúa-Ortega et al. [51] and Ávila-Pérez et al. [53], such values can be referred to as a terrigenous origin, likely related to a release from soils or their resuspension. Slightly enriched (2 ≤ EF ≤ 6) samples are 1C for Hg, 1U for As, and 2U for Sb, probably due to the emission from the nearby area of the furnaces’ edifice (Figure 1). It is also worth noting that the high concentrations of Sb and As in the soil within the mining area and close to 1 and 2 sites, i.e., up to 891 and 251.6 mg/kg (Meloni et al., unpublished data) and higher values than those reported by [54], respectively, likely contribute to the observed EF values, as well as to the highly Sb-enriched 1U sample, through soil remobilization processes. On the contrary, all the uncovered mosses are strongly enriched in Hg (EF > 10).

**Figure 6:**
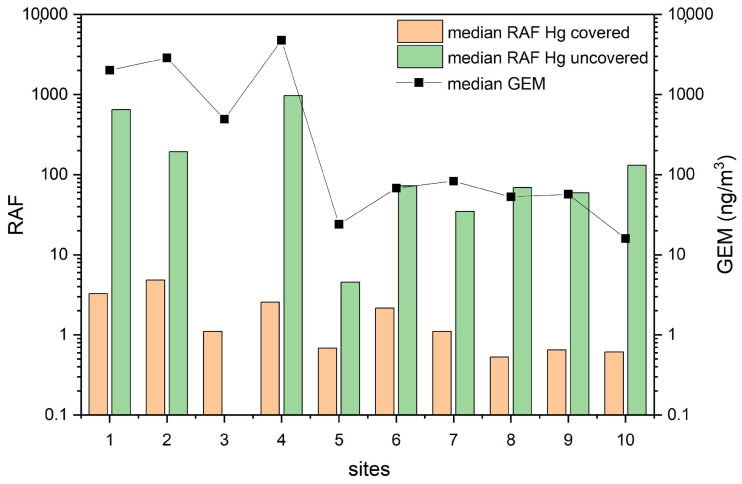
Median of RAF Hg values in covered and uncovered mosses and median of GEM (in ng/m^3^) in the 10 sampling sites. See the text for further details.

The Hg accumulation of the mosses is intimately consistent with the measured, though very limited in time, GEM concentrations (Figure 6), the highest values being measured at sites 1, 2, and 4 (14,761, 18,594, and 19,927 ng/m^3^, respectively; Table 3). These contents exceed both the guideline value for annual average chronic exposure to inorganic Hg vapor (1000 ng/m^3^; [7]), as well as, along with sites 3 and 7 (Table 3), the outdoor limit value recommended by local regulations (300 ng/m^3^; Regional Decree No. 1447, November 23, 1998). The measured GEM values agree with those reported by Vaselli et al. [26,27] for the same area. Thus, both the Hg levels in the air and in the mosses undoubtedly depended on the continuous input of Hg into the atmosphere originating from the multiple sources related to the remediation area, i.e., abandoned buildings next to which the moss bags were placed and/or the nearby heterogeneous soils where waste material from cinnabar processing was deposited in the past [26,54], as well as on the frequent interventions related to the ongoing reclamation [27]. It is worthwhile to mention that liquid mercury below the condensers is still dripping, likely contributing to the anthropogenic GEM captured by mosses [55]. In this regard, mosses at site 3 were less susceptible to the continuous Hg input (despite the lack of uncovered data), as confirmed by the relatively lower GEM concentrations (max ~2000 ng/m^3^; Table 3) and lower RAF values than 1, 2, and 4 sites.

All the evidence so far reported suggests the involvement of both dry and wet deposition processes on the exposed moss bags. For the uncovered samples affected by both depositions, the higher RAF and EF values and the good correlation between the three analyzed elements indicate that wet deposition is a key factor for determining their accumulation on mosses. Nevertheless, direct dry deposition is still an important transport process, especially where a constant and abundant source of Hg, as well as of As and Sb, exists. This is particularly evident for the covered moss bags located within the mining area. Furthermore, it explains the low correlation between covered and uncovered mosses for both As and Sb (Figure 4). As Hg is concerned, whose principal source is the ASS former mining area, e.g., [26,54], it can be emphasized that GEM is probably captured from the atmosphere, due to the high specific surface of mosses, by both dry and wet deposition processes [56]. GEM uptake in mosses is governed by several functional groups in their body surface [57]: once adsorbed, Hg^0^ is promptly oxidized into low-mobility Hg^2+^ thanks to cells catalase activity, and thus becoming strongly bound to the moss with almost no loss for several weeks [58,59]. Since the moss bags were installed during wintertime with relatively low temperatures, it is also likely that post-deposition volatilization processes did not affect the gaseous Hg adsorbed by mosses, e.g., [60]. Nevertheless, the difference in terms of Hg concentration between covered and uncovered mosses might be explained by a larger contribution of wet deposition that influenced the uncovered mosses. From this point of view, despite sequential withdrawal and analysis of the mosses not performed in this study, we can suppose that the bags strongly accumulate Hg in the first period of exposure, as already observed in other studies [61,62]. This hypothesis stems from the rainfall records (Figure 3): in fact, in the first week of exposure, the precipitation reached the highest values of the entire study period, suggesting a great contribution of wet deposition to the Hg concentrations measured in the uncovered moss bags. Precipitation via raindrops and uptake of particulate Hg by mosses, e.g., [63,64] is likely, the latter persisting in the air with GEM, e.g., [65] and being able to adhere to the spongy structure of mosses. Sakata and Asakura [66] indeed suggested that Hg wet deposition depends on the scavenging by precipitation of both divalent reactive gaseous mercury (RGM) and atmospheric particulate mercury (Hg_p_). Di Palma et al. [67] demonstrated that there is a close association and a proportional increase between the amount of particulate diffused in the area under remediation and the moss element uptake. Berg and Steinnes [68] found significant relationships between metal concentrations in moss and wet deposition. Moreover, moss permanent, though not intense, watering increases cell walls permeability, and thus the accessibility of metal ions to ion exchange functional sites [69], while the water-soluble elements of the deposited particles become more available for absorption [70]. Moss species are considered particularly efficient heavy metal adsorbers due to their leaf structure [58]. In particular, the used moss species (*Sphagnum*) is highly capable of absorbing water and keeping it within its large cells [70].

Sites 5 and 10 (Figure 1), located far from the former mining area, had the lowest Hg concentrations as well as the lowest GEM values (Table 3 and Figure 6). The low EF values of Hg, As, and Sb (Table 5), as well as the low RAF values (Table 4 and Figure 6) of site 5, are indeed in agreement with a greater distance from the source of contamination. On the contrary, site 10, despite being in a forest at the highest altitude among the selected points, was characterized by relatively high uncovered RAF values and, apart from the 1, 2, and 4 sites, among the highest EF values (Table 5). In this case, the wet deposition process, besides rain, might also be associated with snowfall, which is rather common at the altitudes they were located, and that can be related to the temperature drop (even below zero) recorded in the final part of the sampling phase (Figure 3). The deposition of Hg under these conditions could be higher since particulate Hg is likely more efficiently scavenged by snow than rain because of its larger surface area [71,72,73]. However, a minor contribution from the local soil cannot be ruled out either, also considering the relatively high Hg concentration recorded in the topsoils collected near the Mt. Amiata summit with respect to other forested areas at low altitudes [74].

Sites 6–7 and 8–9 (Figure 1) were positioned near the Ermeta and Acquapassante chimneys, respectively. GEM concentrations, although lower than those measured inside the former ASS mine, were nevertheless relatively high (Table 3 and Figure 6). In fact, it must be considered that the two chimneys are intercepting a CO_2_-rich fertile horizon at >100 m depth, and the related gases (including GEM) are continuously discharging into the atmosphere, causing severe worsening of the local air quality [35]. Sites 7 and 9, being relatively close to the two chimneys, were more easily affected by the released gases, as confirmed by the higher GEM values with respect to those registered at sites 6 and 8, respectively (Table 3). Mosses were also affected by this release, as confirmed by EF values, especially regarding Hg and the uncovered mosses (Table 5), whose greater accumulation (especially at site 6) could be again explained by a larger contribution from wet deposition (through rain and/or snow).

## 6. Conclusions

The results of the active biomonitoring survey revealed that non-indigenous moss bags are an extremely useful tool for understanding the way Hg can be dispersed, transported, and accumulated. In this study, the main Hg emissions sources in Mt. Amiata were recognized and associated with the former mining area of Abbadia San Salvatore and the Ermeta and Acquapassante chimneys. This was also confirmed by the measured GEM concentrations near the moss bag sites at the beginning of biomonitoring. The behavior of As and Sb accumulated by mosses was different. The latter two are likely related to the soil cover enriched in As-Sb-rich post-roasting waste material. The good correlation between the three analyzed elements, as well as the high RAF and EF values, indicates that wet deposition is a key factor for their accumulation on the uncovered mosses, with a likely strong accumulation in the first heavy-rain period of exposure, while dry deposition is still important for the covered samples, especially those located within the mining area (from 1 to 4). Once adsorbed, GEM strongly binds to the moss through oxidation with almost no loss and without post-deposition volatilization thanks to the low temperatures, but precipitation via raindrops and uptake of particulate Hg by mosses is likely, as favored by *Sphagnum* high water absorption and storage capacity. Moreover, for some sites at higher altitudes (from 6 to 10), even snowfall may have contributed to the mosses’ accumulation thanks to a more efficient scavenging of particulate Hg.

The complementary use of the moss bags technique and of continuous and real-time measurements by dedicated instrumentation for Hg is therefore functional to recognize the dispersion of this contaminant and could be implemented by a greater diffusion over the territory of the mosses, to be then subjected to sequential sampling and subsequent analysis. Repetition over time of continuous measurements by Lumex device around the moss bags sites is expected to highlight local and/or seasonal variations.

## Figures and Tables

**Table 1:** Fixed points, geographical position (coordinates in UTM WGS84), altitude (in meters), and mosses exposure time (in days).

Sampling Site	Coordinates	Altitude	Location	Exposure Time
1	32 T 717,627 4,751,082	883	ASS former mining area, Unit 6	~61
2	32 T 717,612 4,751,045	883	ASS former mining area, Unit 6	~61
3	32 T 717,501 4,751,191	898	ASS former mining area, Unit 6	~61
4	32 T 717,456 4,751,211	908	ASS former mining area, Unit 6	~61
5	32 T 718,255 4,750,750	842	ASS tennis club	~61
6	32 T 716,157 4,751,463	1085	Ermeta chimney	~60
7	32 T 716,125 4,751,378	1082	Ermeta chimney	~60
8	32 T 716,527 4,752,534	1058	Acquapassante chimney	~60
9	32 T 716,552 4,752,465	1062	Acquapassante chimney	~60
10	32 T 715,116 4,753,543	1285	Primo Rifugio Amiatino	~60

**Table 2:** Moss IDs, minimum (min), median, mean, maximum (max), standard deviation (SD) values of Hg (in ng/g), and concentrations of As and Sb (in ng/g) in the mosses. For Hg, 5 replicates of each sample were performed. See the text for further details.

Moss ID	Hg					As	Sb
	Min	Median	Mean	Max	SD		
blank	64	85	91	130	28	59	13
1C	323	363	369	421	49	307	49
2C	277	497	476	653	154	72	23
3C	151	179	179	207	39	87	34
4C	269	303	355	463	91	164	384
5C	134	143	143	153	13	95	16
6C	205	268	257	299	48	76	15
7C	163	179	179	196	24	84	17
8C	101	130	122	136	19	130	26
9C	132	140	140	148	11	89	23
10C	131	137	137	144	8.8	91	38
1U	54,092	55,186	55,186	56,279	1546	2147	6243
2U	16,003	16,523	16,523	17,043	735	761	3439
3U	/	/	/	/	/	/	/
4U	30,142	82,317	75,893	133,842	37,674	528	796
5U	209	473	535	923	280	84	59
6U	4046	6258	6560	9197	2474	445	465
7U	1365	3042	2744	4238	1190	118	205
8U	3634	5960	5647	7036	1678	1370	500
9U	3441	5128	4805	5990	1241	725	754
10U	9498	11,203	11,162	12,776	1310	486	638

**Table 3:** Fixed point, number of measurements for each point (N), and minimum (min), median, mean, maximum (max), and standard deviation (SD) values of GEM (in ng/m^3^).

Sampling Site	GEM					
	N	Min	Median	Mean	Max	SD
1	612	160	2018	3467	14,761	3500
2	453	709	2861	3927	18,594	2979
3	691	281	495	662	2154	377
4	783	47	4785	5325	19,927	3589
5	480	19	24	24	39	2.6
6	721	53	68	75	105	15
7	648	67	83	200	1302	278
8	353	17	53	50	90	20
9	386	24	57	57	122	20
10	571	10	16	17	31	3.0

**Table 4:** Moss IDs and Hg, As, and Sb RAF values. See the text for further details.

Moss ID	RAF Hg	RAF As	RAF Sb
1C	3.3	4.2	2.8
2C	4.8	0.2	0.8
3C	1.1	0.5	1.6
4C	2.6	1.8	29
5C	0.7	0.6	0.2
6C	2.2	0.3	0.1
7C	1.1	0.4	0.3
8C	0.5	1.2	1.0
9C	0.6	0.5	0.8
10C	0.6	0.5	1.9
1U	648	35	479
2U	193	12	264
3U	/	/	/
4U	967	7.9	60
5U	4.6	0.4	3.5
6U	73	6.5	35
7U	35	1.0	15
8U	69	22	37
9U	59	11	57
10U	131	7.2	9.8

**Table 5:** The Enrichment Factor (EF) of Hg, As, and Sb in the mosses. See the text for further details.

Moss ID	Hg EF	As EF	Sb EF
1C	2.23	0.47	0.63
2C	0.82	0.03	0.08
3C	0.77	0.09	0.31
4C	0.41	0.05	1.08
5C	0.35	0.06	0.08
6C	2.0	0.19	0.32
7C	1.12	0.13	0.22
8C	0.66	0.16	0.27
9C	0.41	0.06	0.14
10C	0.86	0.14	0.49
1U	103,504	4.03	11.7
2U	27,847	1.28	5.80
3U	/	/	/
4U	48,227	0.31	0.47
5U	702	0.12	0.09
6U	9792	0.70	0.73
7U	3314	0.13	0.22
8U	1716	0.39	0.14
9U	2835	0.40	0.42
10U	16,125	0.70	0.92

## Data Availability

The data presented in this study are available in the article and Supplementary Materials.

## Supplementary Materials

The following supporting information can be downloaded at: https://www.mdpi.com/article/10.3390/toxics13010002/s1, Table S1: Maximum, minimum, and mean temperature (°C) and precipitation (mm) daily data from the ASS weather station TOS07000001 (Tuscany Regional Hydrological Service, www.sir.toscana.it) during the study period.
