# Supplementary material for: Active Moss Biomonitoring of Mercury in the Mine-Polluted Area of Abbadia San Salvatore (Mt. Amiata, Central Italy)
Source: Toxics. 2024 Dec 24;13(1):2. doi: 10.3390/toxics13010002 (PMC11768723; doi:10.3390/toxics13010002)
Supplement: Supplementary file 1 [file toxics-13-00002-s001.zip › toxics-3354715-supplementary.pdf]

Supplementary Table S1: Maximum, minimum and mean temperature (° C) and precipitation (mm) daily data from the ASS weather station TOS07000001 (Tuscany Regional Hydrological Service, www.sir.toscana.it) during the study period.

Date	T max [° C]	T min [° C]	T mean [° C]	Precipitation [mm]
03/10/2013	17.5	7.5	12.7	0
04/10/2013	18.1	11.2	14.3	0
05/10/2013	16.4	12.2	14.4	32
06/10/2013	18.9	11.8	14.3	72
07/10/2013	17.9	11.4	13.5	7
08/10/2013	15.6	10.5	12.4	8
09/10/2013	16.4	9.4	12.3	8
10/10/2013	15.3	9.3	12.4	0
11/10/2013	16.4	8.3	11.9	23
12/10/2013	16.2	8.8	12.0	15
13/10/2013	18.2	8.5	12.6	0
14/10/2013	20.4	9.7	14.2	0
15/10/2013	19.9	11.0	13.8	0
16/10/2013	17.4	12.8	14.5	3
17/10/2013	19.8	10.3	14.6	0
18/10/2013	18.7	9.9	14.0	0
19/10/2013	18.8	10.9	14.3	0
20/10/2013	17.1	10.9	14.3	0
21/10/2013	16.4	12.6	14.8	1
22/10/2013	19.6	11.7	15.5	27
23/10/2013	18.2	13.3	15.4	0
24/10/2013	16.8	12.4	14.5	19
25/10/2013	19.4	11.4	14.6	3
26/10/2013	19.4	11.6	15.0	0
27/10/2013	18.8	10.6	14.6	0
28/10/2013	19.0	11.5	15.1	0
29/10/2013	20.6	11.1	15.3	0
30/10/2013	18.0	8.9	13.5	0
31/10/2013	15.7	10.5	12.8	0
01/11/2013	16.7	9.8	12.9	0
02/11/2013	15.4	10.8	12.6	2
03/11/2013	14.5	11.7	13.3	6
04/11/2013	15.8	10.0	12.0	1
05/11/2013	11.7	8.6	10.0	19
06/11/2013	15.6	8.3	11.7	0
07/11/2013	16.9	8.9	12.3	0
08/11/2013	16.0	9.7	13.5	0
09/11/2013	15.5	10.5	13.3	1
10/11/2013	12.5	5.7	9.6	0
11/11/2013	6.1	2.4	4.6	26
12/11/2013	9.6	6.1	7.8	2

13/11/2013	12.0	8.2	9.5	0
14/11/2013	14.6	7.7	10.6	0
15/11/2013	11.1	7.2	9.2	2
16/11/2013	10.6	5.8	8.5	0
17/11/2013	10.5	5.8	8.7	0
18/11/2013	11.9	7.9	9.7	0
19/11/2013	11.3	5.8	7.7	9
20/11/2013	10.8	4.3	6.6	10
21/11/2013	6.3	1.1	3.6	2
22/11/2013	6.2	1.4	3.4	22
23/11/2013	4.0	1.7	2.6	10
24/11/2013	6.4	1.4	3.9	7
25/11/2013	5.5	-3.2	1.6	0
26/11/2013	0.4	-3.8	-2.2	1
27/11/2013	-0.1	-4.2	-2.3	0
28/11/2013	3.3	-2.1	-0.2	0
29/11/2013	8.3	-0.3	3.1	0
30/11/2013	7.2	-0.3	2.8	0
01/12/2013	4.4	2.7	3.4	0
02/12/2013	5.2	2.7	4.1	0
03/12/2013	8.4	2.5	5.0	0
